# Case Report: Mosaicism of a novel nonsense variant in the neurofibromin gene underlies a mosaic generalized NF1 phenotype

**DOI:** 10.12688/f1000research.28052.2

**Published:** 2021-06-30

**Authors:** Hui Li Kwong, Yong-Kwang Tay, Ene-Choo Tan

**Affiliations:** 1 Changi General Hospital, Singapore, Singapore; 2 KK Women's and Children's Hospital, Singapore, Singapore; 3 Paediatrics Academic Clinical Programme, SingHealth Duke-NUS Medical School, Singapore, Singapore

**Keywords:** mosaicism, mutation, neurofibromatosis 1, sequencing

## Abstract

Neurofibromatosis 1 (NF1) is a neurocutaneous syndrome characterized by multiple café-au-lait macules, cutaneous neurofibromas or plexiform neurofibromas, iris Lisch nodules, axillary and inguinal freckling. Mosaicism in NF1 can either present as a generalized disease, or in a localized (segmental) manner. Mosaic generalized NF1 may have presentations that are similar to generalized NF1 or have a milder phenotype and hence may be under-recognised in clinical practice. We report a nonsense mutation in the *NF1* gene in a 55-year old Chinese male with the mosaic generalized phenotype. He reported noticing increasing numbers of skin-colored papules over his face, neck, back and abdomen when he was about 40 years old. From both next-generation and Sanger sequencing data, the variant appeared to be mosaic and present at about 24%. It is in exon 39 and has not been reported in any database or published literature.

## Introduction

Neurofibromatosis 1 (NF1, OMIM#162200) is a neurocutaneous syndrome characterized by café-au-lait macules, cutaneous neurofibromas or plexiform neurofibromas, iris Lisch nodules, axillary and inguinal freckling ^1^. Other features include optic nerve gliomas, central nervous system tumours, and long bone deformities. The autosomal dominant condition is caused by mutations or deletions involving the neurofibromin 1 gene ( *NF1*, OMIM*613113) at chromosome 17q11.2, one of the largest human genes (282 kb) with 57 exons and three alternatively spliced transcripts ^2^. Over 3600 clinically significant *NF1* variants are documented in the Human Gene Mutation Database (HGMD). They are distributed over the whole gene from the first to the last exon.

The estimated incidence of germline NF1 is 1:2500–3000, with approximately half due to *de novo* mutations and the other half inherited from an affected parent. The disease is highly penetrant and although the offspring are at 50% risk of inheriting a mutation, the manifestations are extremely variable even for family members who share the same mutation ^1^. Non-germline or mosaic NF1 is rare at 1:36,000–40,000, although it is probably under-reported as most cases are diagnosed clinically and molecular confirmation is not always performed ^3^.

## Case report

We report a patient with adult-onset NF1 presentations. He is of Chinese ancestry and the parents are not consanguineous. The 55-year-old male engineer presented in 2015 with increasing numbers of skin-colored papules over the past 13 years. The patient reports the onset of numerous light-brown macules in his late teens, starting on his trunk and back. He did not recall having these lesions earlier in life. The neurofibromas were only noted to occur in his 30s. Clinically he has generalized small 1 – 5 mm light-brown macules on his trunk and back (not limited to a segment or blaschkoid distribution), the largest of which measured approximately 10 mm. Excision of one such representative lesion measuring 0.5 mm × 0.5 mm from the right lateral neck was consistent with a neurofibroma. He was referred to dermatology and neurology departments on suspicion of neurofibromatosis.

Clinically the patient had more than 20 small, soft, flesh-coloured papules and small nodules (ranging between 4 and 10 mm) on his face, neck, trunk and limbs. There was no axillary or inguinal freckling. He also had multiple scattered café-au-lait macules on his trunk. There were no lesions to suggest plexiform neurofibromas and no clinical evidence of long bone deformities. Slit-lamp examination revealed the presence of Lisch nodules in both irises. He had three older siblings, none of whom had similar skin lesions. Neither his parents nor members of his extended family had any features of neurofibromatosis. His two daughters (aged 17 and 21 years) were well with no features of neurofibromatosis. His other medical history was only significant for hyperlipidaemia for which he was on simvastatin (20 mg nightly) and cervical spondylosis which did not require intervention and he is no longer on active follow-up with the orthopaedics department.

**Figure 1.**
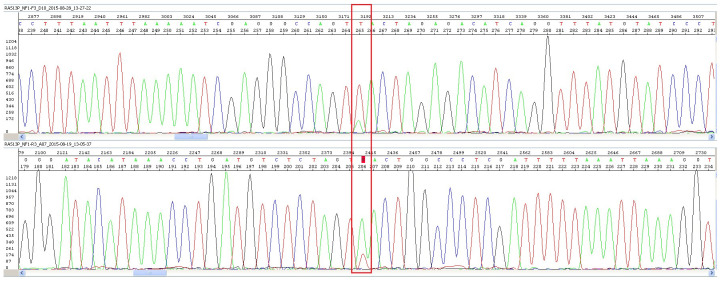
Chromatogram from Sanger sequencing for the c.5612T>A variant using forward primer (top) and reverse primer (bottom). Shown is the wildtype T allele with a small peak for the variant allele A (rectangular box).

Approval for sequencing analysis was obtained from the SingHealth Institutional Review Board (CIRB Ref 2014/207/F). Venous blood was collected from the patient after written informed consent and genetic counselling. Next-generation sequencing on genomic DNA was performed using a customised targeted panel (which covered all *NF1* exons and *NF1*-related genes) with the Agilent Haloplex Target Enrichment System (Agilent Technologies, Santa Clara, USA) and the MiSeq System (Illumina, San Diego, USA). Output reads were processed using the MiSeq Reporter pipeline and variants were annotated with ANNOVAR (2019Oct24) ^4^. A heterozygous single nucleotide substitution in the *NF1* gene that is predicted to result in premature termination: NM_000267.3: 5612T>A (p.L1871X) was identified. The number of reads for the variant was 28 out of 117. The presence of the variant was confirmed in our laboratory by Sanger sequencing using BigDye Terminator v3.1 in a volume of 10 µl. Amplification conditions included initial denaturation at 96°C for 1 min, followed by 25 cycles of 96°C for 10 sec, 50°C for 5 sec; and 60°C for 4 min. Following ethanol purification, the products were resuspended in Hi-Di™ formamide and loaded onto the ABI 3130 Genetic Analyzer (Applied Biosystems, Foster City, USA). The peak height of the variant was approximately one-quarter to one-third that of the wild-type for both forward and reverse primers, confirming the mosaicism ( Figure 1).

The variant identified has not been reported in any population databases and is not found in ClinVar or the Human Gene Mutation Database. However, a missense variant involving the same codon changing it to a phenylalanine residue has been reported in a patient with NF1 ^5^. A search of the ClinVar database found several variants within 10 nucleotides identified from patients with NF1 or hereditary cancer-predisposing syndrome, indicating that this small region might be more prone to spontaneous mutations ( Table 1).

**Table 1.** ClinVar record of variants within 10 nucleotides of the identified c.5612T>A variant.

Accession	Variant	Protein change	Transcript ID	Condition	Interpretation ^#^
VCV000663647	5605G>T	G1869C	NM_000267.3	NF1	Uncertain significance
VCV000578661	5606G>T	G1869V	NM_000267.3	NF1	Uncertain significance
VCV000654611	5606_5627del	G1869fs	NM_000267.3	NF1	Pathogenic
VCV000237577	5608C>T	Q1870*	NM_000267.3	NF1	Pathogenic
VCV000431662	5609dup	L1871fs	NM_000267.3	NF1	Pathogenic
VCV000457758	5610G>A	Q1870*	NM_000267.3	NF1	Likely benign
VCV000825851	5673G>T	Q1870H	NM_001042492.3	HCPS	Uncertain significance
VCV000547665	5613A>C	L1871F	NM_000267.3	NF1	Uncertain significance
VCV000547666	5613dup	L1872fs	NM_000267.3	NF1	Pathogenic
VCV000229667	5613A>G	L1871=	NM_000267.3	HCPS	Likely benign
VCV000957518	5672_5673ins	L1871fs	NM_001042492.3	NF1	Pathogenic
VCV000653673	5617G>T	E1873*	NM_000267.3	NF1	Pathogenic
VCV000457759	5617G>C	E1873Q	NM_000267.3	NF1	Uncertain significance

HCPS, Hereditary cancer-predisposing syndrome; NF1, Neurofibromatosis Type 1. ^#^Interpretation is based on submission in ClinVar.

## Discussion

Mosaic NF1 was previously known as segmental NF1. It was initially defined as clinical features of NF1 limited to one or more segments of the body. The mosaicism which is caused by postzygotic mutations in the *NF1* gene in somatic cells can present as a generalized disease or in a localized (segmental) manner ^6, 7^. Mosaic generalized patients may be clinically indistinguishable from generalized NF1 patients or have a milder phenotype ^8^. In contrast, the cutaneous manifestations of mosaic localized NF1 reflect the pattern of skin mosaicism and can vary from a narrow strip, one-quadrant, or half the body, unilaterally or bilaterally, in a symmetrical or asymmetrical fashion. Mosaic-localized NF1 patients may develop only pigmentary changes or neurofibromas, have a combination of pigmentary and neurofibromas, or have plexiform neurofibromas only ^7, 9, 10^.

Like generalized NF1, mosaic NF1 presents with pigmentary features and plexiform neurofibroma arising in childhood, and dermal neurofibromas usually in adulthood. However, not all patients with mosaic NF1 with pigmentary changes develop neurofibromas. Patients may also develop learning difficulties, pseudoarthrosis and malignancy, despite having a milder phenotype ^3, 10, 11^. Our patient is cognitively normal and had noticed the development of neurofibromas while in his late thirties. He also had Lisch nodules, which were not frequently observed regardless of the patient's age in other cohorts ^3, 6, 12^. He is currently on annual follow-up for his cutaneous presentations.

Most cases of mosaic NF1 did not have molecular confirmation of the presence of an *NF1* mutation, but genetic analysis was performed in a case series of eight patients with segmental NF1 from Canada. Sequencing of DNA from skin lesions revealed a pathogenic loss-of-function variant in the *NF1* gene in all eight: six were single or multi-nucleotide variants, while the other two were microdeletions. Seven out of the eight patients had pigmentary changes (cafe-au-lait macules and axillary freckling) ˗ with three of them having non-localized lesions and one with lesions crossing the midline. The eighth patient had cafe-au-lait macules, a plexiform neurofibroma and sphenoid wing dysplasia but no freckling. Only one patient had Lisch nodules ^12^.

The risk of passing on the full-blown or localized disease to future generations is 50% for those with germline mutations. Although gonadal mosaicism is rare, individuals with mosaic *NF1* mutation may transmit the mutation to their offspring in such cases ^3, 13^. Thus, patients with mosaic NF1 should be counselled regarding the possibility of gonadal NF1 mosaicism and the risk of transmission to the next generation, especially when the mutation is also present in non-dermal tissues. Our patient had 24% mosaicism based on the number of reads from next-generation sequencing results of DNA from his peripheral blood. He was not evaluated for somatic or gonadal mosaicism. His two daughters appear to have no features of NF1, although he is considering genetic testing for his children.

In summary, we report a novel nonsense mutation in the *NF1* gene, resulting in a mosaic generalized phenotype of NF1. Studies have sought to examine the genotype-phenotype correlation in mosaic localized NF1, although there is little in the literature regarding mosaic generalized NF1, which may be under-recognized given the clinical similarities with classic NF1. Future genotype-phenotype correlation studies in mosaic generalized NF1 would lead to a better understanding of the disease development process and long-term outcomes. With better sequencing technology that can detect low-level mosaicism, molecular confirmation should be carried out to enable healthcare professionals to provide a more accurate prognosis and genetic counselling.

## Data availability

ClinVar accession number: SCV001450721.

## Consent

Written informed consent for publication of the clinical details was obtained from the patient.
