# Supplementary material for: Case Report: Mosaicism of a novel nonsense variant in the neurofibromin gene underlies a mosaic generalized NF1 phenotype
Source: F1000Res. 2021 Jun 30;10:148. Originally published 2021 Feb 25. [Version 2] doi: 10.12688/f1000research.28052.2 (PMC8215559; doi:10.12688/f1000research.28052.2)
Supplement: Peer review history

# Peer review: Reviewer response for version 1

**DOI:** 10.5256/f1000research.31028.r83797

**Published:** 2021-06-18

**Authors:** Joshua Tay

**Affiliations:** 1 Department of Otolaryngology-Head and Neck Surgery, National University of Singapore, Singapore, Singapore

This is a well written article demonstrating a thorough genomic evaluation of the NF1 gene in an adult-onset NF1 patient. The NF1 mutation identified was present on both NGS (23.9%) and Sanger sequencing (1/3 to 1/4) at similar variant allele frequencies, supporting the mosaicism.

 Mosaic generalized NF1 is less commonly seen in the clinic compared to its well-known, germline NF1 counterpart. I agree with the authors that this could also be a result of an under-recognition of mosaic cases, given their clinical similarities. NGS diagnostics and recognizing the importance of variant allele frequencies, like those presented here, provides an opportunity to close this gap.

Are enough details provided of any physical examination and diagnostic tests, treatment given and outcomes?

Yes

Is the case presented with sufficient detail to be useful for other practitioners?

Yes

Is sufficient discussion included of the importance of the findings and their relevance to future understanding of disease processes, diagnosis or treatment?

Yes

Is the background of the case’s history and progression described in sufficient detail?

Yes

Reviewer Expertise:

I am an Otolaryngology surgeon with a PhD in Cancer Biology, focusing on cancer genomics and molecular subtypes of disease.

I confirm that I have read this submission and believe that I have an appropriate level of expertise to confirm that it is of an acceptable scientific standard.

# Peer review: Reviewer response for version 1

**DOI:** 10.5256/f1000research.31028.r80275

**Published:** 2021-03-15

**Authors:** Richard J. Antaya

**Affiliations:** 1 Departments of Dermatology and Pediatrics, Yale University School of Medicine, New Haven, CT, USA

This is an enlightening and well written case report that brings to light a variant of a common genetic syndrome.

1. In the introduction the authors state that that non-germline or mosaic NF-1 is very rare, however they report that it is about 10 times less common than germline NF-1, which is actually relatively common. I would agree it is rare, but not very rare. The statement that many cases of generalized mosaic NF1 are likely undiagnosed is likely true and would further decrease the scarcity of this disorder.
2. In the description of the case, the authors state that the patient had café au lait macules (CALMs) on his trunk, but do not state the size, number, and especially the duration of these lesions. In germline NF-1 CALMs generally precede the development of neurofibromas by often a decade or more. I think this is important information to include for the readership.
3. I feel that only gonadal mosaicism should be used in this discussion since the term  gonosomal is relating primarily to sex chromosomes, and ch17, the chromosome with the mutation of interest, is an autosome.
4. While not clear from the discussion, have any studies evaluated the percent mutated cells in somatic tissue, skin versus blood, in patients with either generalized or segmental mosaic NF1. Since patients with cutaneous “segmental” NF1 are known to harbor the mutation in other tissues and pass on the disease to offspring, do these individuals have a form or generalized mosaic NF1 or is it tissue/area specific? I understand if this info is not available.

Are enough details provided of any physical examination and diagnostic tests, treatment given and outcomes?

Yes

Is the case presented with sufficient detail to be useful for other practitioners?

Yes

Is sufficient discussion included of the importance of the findings and their relevance to future understanding of disease processes, diagnosis or treatment?

Yes

Is the background of the case’s history and progression described in sufficient detail?

Partly

Reviewer Expertise:

I am an academic pediatric dermatologist with a cursory understanding of the details regarding the methodology of genetic mutation analysis but am very familiar with the cutaneous manifestations of NF1 and segmental NF1.

I confirm that I have read this submission and believe that I have an appropriate level of expertise to confirm that it is of an acceptable scientific standard.

# Response

**Published:** 2021-06-23

**Authors:** Ene Choo Tan

**Affiliations:** KK Women's & Children's Hospital, Singapore

We have deleted the word "very" from "very rare" in the Introduction. We also added more details about the patient, including his ethnicity and that his parents are not consanguineous, and the size and duration of his lesions. We also changed "gonosomal" to "gonadal" as suggested.
